# Intestinal Microbiota Reduction Followed by Fasting Discloses Microbial Triggering of Inflammation in Rheumatoid Arthritis

**DOI:** 10.3390/jcm12134359

**Published:** 2023-06-28

**Authors:** Thomas Häupl, Till Sörensen, Biljana Smiljanovic, Marine Darcy, Justus Scheder-Bieschin, Nico Steckhan, Anika M. Hartmann, Daniela A. Koppold, Bruno Stuhlmüller, Karl Skriner, Barbara M. Walewska, Berthold Hoppe, Marc Bonin, Gerd R. Burmester, Pascal Schendel, Eugen Feist, Karsten Liere, Martin Meixner, Christian Kessler, Andreas Grützkau, Andreas Michalsen

**Affiliations:** 1 Department of Rheumatology and Clinical Immunology, Charité—Universitätsmedizin Berlin, Corporate Member of Freie Universität Berlin and Humboldt-Universität Berlin, 10117 Berlin, Germany; 2 Institute of Social Medicine, Epidemiology and Health Economics, Charité—Universitätsmedizin Berlin, Corporate Member of Freie Universität Berlin and Humboldt-Universität Berlin, 10117 Berlin, Germany; 3 Department of Rheumatology, Helios Fachklinik Vogelsang-Gommern GmbH, 39245 Gommern, Germany; 4 Department of Dermatology, Venereology and Allergology, Charité—Universitätsmedizin Berlin, Corporate Member of Freie Universität Berlin and Humboldt-Universität Berlin, 10117 Berlin, Germany; 5 Department of Internal and Integrative Medicine, Immanuel Hospital Berlin, 14109 Berlin, Germany; 6 Department of Pediatrics, Division of Oncology and Hematology, Charité—Universitätsmedizin Berlin, Corporate Member of Freie Universität Berlin, Humboldt-Universität Berlin and Berlin Institute of Health, 10117 Berlin, Germany; 7 Institute of Laboratory Medicine, Unfallkrankenhaus Berlin, 12683 Berlin, Germany; 8 Amedes Genetics, 10117 Berlin, Germany; 9 Services in Molecular Biology GmbH, 10115 Rüdersdorf, Germany; 10 Deutsches Rheuma-Forschungszentrum, 10117 Berlin, Germany

**Keywords:** rheumatoid arthritis, fasting, monocytes, cytometric profiling, intestinal microbiota, mucosal barrier, dysbiosis

## Abstract

Rheumatoid arthritis (RA) synovitis is dominated by monocytes/macrophages with inflammatory patterns resembling microbial stimulation. In search of triggers, we reduced the intestinal microbiome in 20 RA patients (open label study DRKS00014097) by bowel cleansing and 7-day fasting (≤250 kcal/day) and performed immune monitoring and microbiome sequencing. Patients with metabolic syndrome (*n* = 10) served as a non-inflammatory control group. Scores of disease activity (DAS28/SDAI) declined within a few days and were improved in 19 of 20 RA patients after breaking the fast (median ∆DAS28 = −1.23; ∆SDAI = −43%) or even achieved remission (DAS28 < 2.6/*n* = 6; SDAI < 3.3/*n* = 3). Cytometric profiling with 46 different surface markers revealed the most pronounced phenomenon in RA to be an initially increased monocyte turnover, which improved within a few days after microbiota reduction and fasting. Serum levels of IL-6 and zonulin, an indicator of mucosal barrier disruption, decreased significantly. Endogenous cortisol levels increased during fasting but were insufficient to explain the marked improvement. Sequencing of the intestinal microbiota indicated that fasting reduced potentially arthritogenic bacteria and changed the microbial composition to species with broader metabolic capabilities. More eukaryotic, predominantly fungal colonizers were observed in RA, suggesting possible involvement. This study demonstrates a direct link between the intestinal microbiota and RA-specific inflammation that could be etiologically relevant and would support targeted nutritional interventions against gut dysbiosis as a causal therapeutic approach.

## 1. Introduction

Diet and fasting can have a great influence on health and disease. The effect can be direct, when mediated by individual food components, or indirect, when the metabolization of food by gut microbes is involved. Such effects have been discussed in various etiologically unexplained diseases like multiple sclerosis [1], Parkinson’s disease [2], atopic dermatitis [3] or rheumatoid arthritis [4]. Composition and function of the gut microbiota is shaped by a complex interplay of individual dietary habits in terms of food composition [5], amount [6] and time of intake [7], as well as factors of stress [8]. Thus, the microbiome could be a crucial link for the influence of nutrition in a variety of chronic conditions, including autoimmune, neurodegenerative or cardiometabolic diseases [9].

Caloric restriction and fasting have been described to influence cellular and molecular responses in metabolic syndrome (MetS), but also in inflammatory diseases like rheumatoid arthritis (RA) [10]. In general, caloric restriction for less than 24 h seems to affect monocytes by blood count and activity [11]. Fasting for 36 h in a mouse model resulted in changes in lymphocyte dynamics of Peyer’s patches and bone marrow [12], supporting the hypothesis that food deprivation can notably affect metabolism and vitality of the gut microbiome with implications for immunological defense.

Therapeutic fasting in RA for at least one week achieves relevant clinical improvement [13]. Subsequent individually tailored diets may maintain the benefits of fasting [14,15] even up to one year [13]. Previous studies on caloric restriction revealed changes in the microbiota and improvement of mucosal integrity [16,17,18,19,20]. Thus, dietary components and/or microbial metabolites may contribute to RA pathology via a disturbed intestinal barrier [21,22,23]. This could explain the dominance of innate immune cells like monocytes and granulocytes in joints during flairs, while the adaptive immunity of anticitrullinated-protein antibodies (ACPA) or rheumatoid factor (RF) remain unchanged regardless of activity [24].

So far, beneficial effects of fasting in RA have mostly been discussed as an effect of metabolic changes in general and in particular the endogenous increase in cortisol [25]. However, previously, we found an innate immune response dominant in RA with an increased turnover of monocytes [26] and transcriptome patterns in RA synovitis that matched best with microbial stimulation of monocytes [27]. If an antimicrobial-like response of monocytes is prominent in the joint, we may hypothesize that this is triggered by local intraarticular antigens. The largest resource for microbial products in humans is the gut. So far, culturable microbes or microbial DNA have rarely been detected in the joints of RA patients. However, relevant pathogens in the gut in RA may shed microbial products into the body that accumulate on the joint matrix and trigger chronic inflammation there [28]. So far, we do not know what microbes to screen for, and there may be more than one particular microbe involved in the pathogenesis of RA. Thus, the indirect way could be a first step towards confirming this hypothesis: to show that removal of the individual microbiota will reduce disease activity along with the dominant immune process in most if not all RA patients.

**Figure 1:**
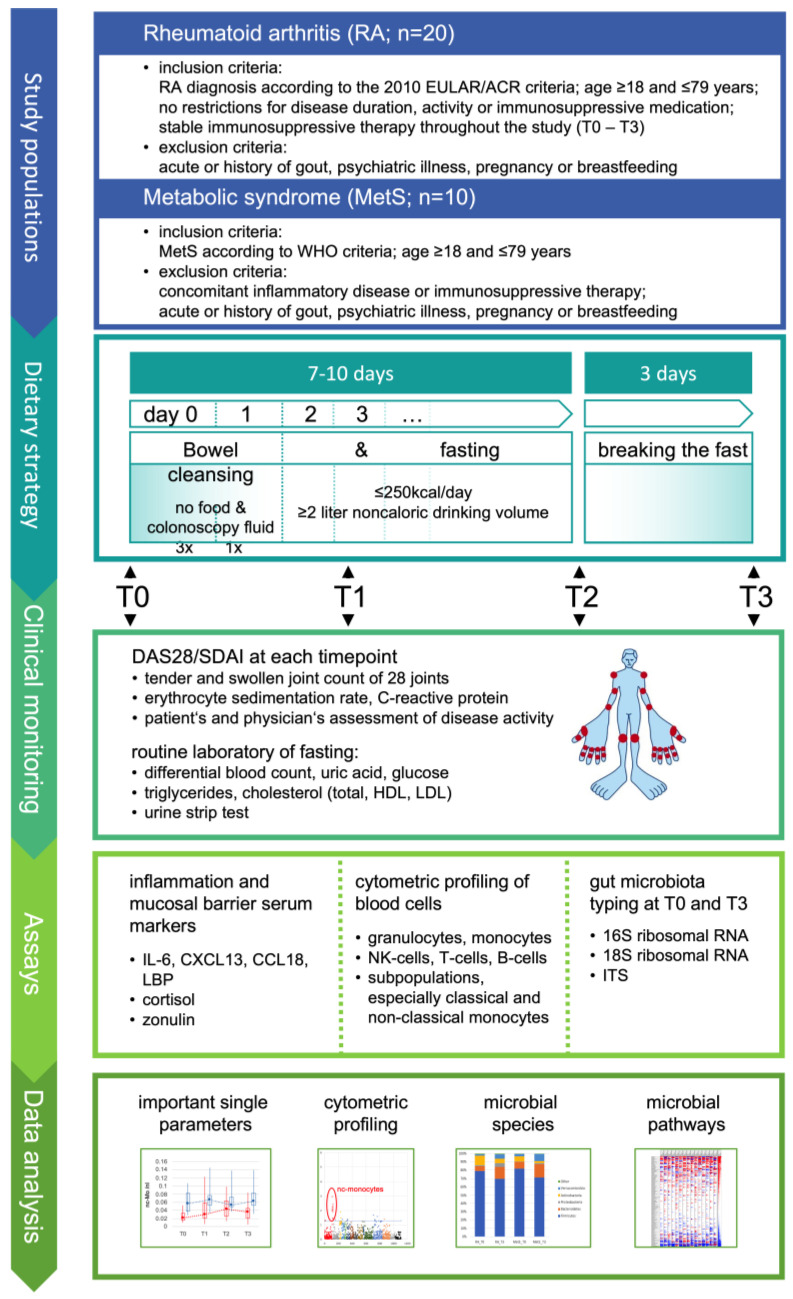
Study design. The study was designed to perform stable reduction in the gut microbiota in rheumatoid arthritis for at least one week and to investigate its effects on subpopulations of immune cells by cytometric profiling and on selected serum parameters as indicated. In parallel, stool samples were collected before microbiota reduction and when re-expanded after fast breaking to characterize the altering gut microbiota for generating further hypotheses. Metabolic syndrome was included as a control to estimate whether significant effects on immune parameters are specific for RA. EULAR: European League Against Rheumatism; ACR: American College of Rheumatology; WHO: World Health Organization; DAS28: disease activity score 28-joint count; SDAI: simplified disease activity index for RA; ITS: nuclear ribosomal internal transcribed spacer; HDL: high-density lipoprotein; LDL: low-density lipoprotein.

We removed the gut microbiota by flushing out more than 95% of the microbial load with colonoscopy fluid [29], optimized by dose splitting [30], and maintained this microbiota reduction by fasting for at least one week. A significant reduction in clinical disease activity was the primary objective. Extensive profiling of leukocyte subpopulations in the blood, serum markers of inflammation and mucosal permeability were performed to identify the dominant immunological changes. The prokaryotic and eukaryotic gut microbiome was investigated as a first pilot study of microbiota reduction in RA. To distinguish RA-specific from general effects, patients with MetS served as a control (Figure 1). The effect of fasting on RA disease improvement is strong and requires only small group sizes for sufficient power. As expected, the primary objective, a reduction in the disease activity score 28-joint count (DAS28) and a simplified disease activity index for RA (SDAI), was achieved, as almost all RA patients improved rapidly. As the dominant effect, and highly significant only in RA, the initially elevated monocyte turnover decreased. Serum levels of interleukin 6 (IL-6) and zonulin also decreased in RA. Analysis of the RA gut microbiota suggested not only prokaryotic but also eukaryotic dysbiosis. In addition, fasting shifted the microbiota towards bacteria with a broader range of metabolic capabilities in both RA and MetS, suggesting opportunities for inducing beneficial changes by fasting in general. The data support microbial triggers in RA, demand further investigation of the gut microbiota, and encourage targeted and personalized nutritional interventions.

## 2. Materials and Methods

### 2.1. Study Objective and Rationale

The aim of the study was to identify key immunological processes involved in the decline in inflammation in RA under conditions of optimized gut microbiota reduction and whether these match with dominant processes of RA pathology. To collect relevant samples, the primary objective was significant reduction in clinical disease activity through microbiota reduction. Secondary objectives were to identify the leading immunological markers. Microbiome analysis was performed as a pilot experiment including prokaryotic and eukaryotic screening, which may provide additional information and orientation for the planning of future studies.

To demonstrate that changes in immunological parameters were not a general effect of microbiota reduction but were specific to RA, MetS patients without inflammatory diseases were used as a reference for non-inflammatory conditions. The study was conducted with inpatients to ensure compliance with the study design. An inpatient study with healthy volunteers was not possible.

The use of randomization and blinding methods was limited, because there are no placebo applications for bowel cleansing and fasting, and neither method can be performed in a blinded manner. To recruit patients in as unbiased a manner as possible, we avoided selection by disease activity or drug treatment and continuously enrolled patients as they came and agreed. Baseline was considered sufficient for assessing the effect of microbiota reduction. It is known from clinical experience that in everyday life, disease activity and routine inflammatory parameters do not improve within a few days if drug treatment is kept stable. Therefore, no additional control group of RA patients was included.

Microbiota reduction can be achieved by bowel cleansing and can be maintained by fasting. In Buchinger fasting, which is an established protocol for fasting in RA, bowel cleansing is involved and performed with Glauber’s salt (sodium sulfate) followed by fasting with less than 250 to 500 kcal per day. However, bowel cleansing exclusively with Glauber’s salt is not optimal, and is no longer used in endoscopic examinations. To reduce the intestinal microbiome as well as possible, intestinal cleansing with colonoscopy fluid was reported to remove more than 95% of the microbiome [29]. In addition, splitting the administration of colonoscopy fluid over 2 days results in less digestive residue in the colonoscopy [30].

### 2.2. Study Design and Population

The study design is outlined in Figure 1. The study was approved by the Charité ethics committee and performed at the Charité Department of Internal and Complementary Medicine in collaboration with the Charité Department of Rheumatology and the Deutsches Rheuma-Forschungszentrum. Written informed consent was obtained from all participants preceding study entry.

Group size calculation for RA patients was based on own observations with Buchinger fasting, which revealed high effects for DAS28 improvement. In the expectation that relevant immunological parameters should perform similarly, the required group size was calculated based on DAS28 criteria using assumptions for paired samples, one-sided *t*-test, effect size ≥ 0.8, alpha = 0.05 and power = 0.95. This resulted in a group size of *n* = 19 RA patients [31]. Thus, we enrolled 20 RA patients diagnosed according to the 2010 European League Against Rheumatism (EULAR)/American College of Rheumatology (ACR) criteria. MetS patients were selected according to WHO criteria. This group was smaller due to limited availability of MetS patients and resources, which was acceptable for us as this group was not relevant for the primary objective.

For inclusion, all participants had to be ≥18 and ≤79 years old and had to apply for fasting in a medically controlled hospital setting. Exclusion criteria were acute or history of gout, psychiatric illness affecting proper consent, pregnancy, or breastfeeding.

Bowel cleansing was initiated with food reduction and was followed by a split dose bowel cleanse (moviprep, Norgine GmbH, Wettenberg, Germany) at day 0 and day 1 and maintained for 7 to 10 days by fasting (<250 kcal per day). In addition, enemas were performed every other day during fasting.

Fast breaking consisted of stepwise slowly increasing amounts of eupeptic vegetarian foods while avoiding sugar. Clinical disease activity (DAS28 and SDAI) served for response assessment [32,33]. Blood analysis was determined in the morning (8 a.m.) at day 0 (baseline; T0), day 3 (early after microbiota reduction, T1), the last day of fasting (stable microbiota reduction, T2) and 3 days after breaking the fast (early expanding gut microbiome, T3).

DAS28 was calculated, including erythrocyte sedimentation rate (ESR) as a blood marker of inflammation, together with the number of tender and swollen joints and global assessment by the patient. DAS28-ESR < 2.6 indicates remission, ≥2.6 and ≤3.2 indicates low, >3.2 and ≤5.1 moderate and >5.1 high disease activity. SDAI was calculated including C-reactive protein (CrP) as blood marker of inflammation, together with the number of tender and swollen joints and global assessment by patients and physician. SDAI up to 3.3 indicates remission, 3.4 to 11 low activity, 11.1 to 26 moderate and 26.1 to 86 high activity. SDAI reduction by >85% indicates major response, >70% and <85% moderate response and >50% and <70% minor response.

Stool samples were collected at T0 and T3, other time points did not reveal representative samples due to bowel cleansing and enemas.

### 2.3. Cytometric Profiling

Cytometric profiling of leukocytes consisted of 10 different cocktails with staining of up to 10 antigens in each cocktail [34]. In total 46 surface antigens were monitored (Table S1). After erythrocyte lysis and staining, cells were fixed with 1% paraformaldehyde and analyzed within 24 h (FACSCanto™ II Flow Cytometer; BD Biosciences, Heidelberg, Germany) with an average cell count of one million cells per sample. BD™ Cytometer Setup and Tracking Beads were regularly used before each measurement and quality of antibody staining was monitored for each individual fluorescence channel for each staining cocktail.

### 2.4. Analysis of Cytometric Data

Using the algorithm immunoClust [34,35], cytometric events were clustered and classified based on uncompensated raw data and without operator-dependent gating. Population clustering and comparative meta-clustering in immunoClust assume finite mixture models and use Expectation-Maximization iterations with an integrated classification likelihood criterion to stabilize the number of reasonable clusters. For meta-clustering, a probability measure on Gaussian distributions was applied, which was based on the Bhattacharyya Coefficients. Meta-clusters were manually annotated and classified (Figure S1). All populations were corrected to cell count per blood volume based on the actual leukocyte blood count of each patient at sampling.

### 2.5. Serum Markers

Serum samples were collected from each RA and MetS patient at T0, T1, T2 and T3. Cytokines were determined by ELISA according to the manufacturer’s protocol: BAFF (Catalog No. DBLYS0B, R&D Systems, Minneapolis, MN, USA); CCL18 (Catalog No. ab100620; abcam, Cambridge, UK); IL-6 (Catalog No. ab46027, abcam, Cambridge, UK); LBP (Catalog No. E-EL-H6108; Elabscience, Houston, TX, USA); Zonulin (Catalog No. PK-EL-K5600; PromoCell, Heidelberg, Germany).

### 2.6. Microbiome Sequencing

Stool samples were collected on the ward, immediately frozen at −70 °C and stored at −20 °C until processing. Complete homogenization of the fecal samples (*n* = 44 from 22 individuals) was achieved by mortaring in liquid nitrogen and the DNA extraction was performed using the *Quick*-DNA^TM^ Fecal/Soil Microbe Miniprep Kit (Catalog No. D6010, Zymo Research, Brühl, Germany) following the instructions of the manufacturer. The extracted genomic DNA was quantified using a Qubit Reader Instrument and the Qubit dsDNA HS (High-Sensitivity) Assay Kit (Thermo Fisher Scientific, Waltham, MA, USA). Then, 100 ng genomic DNA of each sample were taken for the amplification of either 16S-, ITS- or 18S- amplicons in a multiplexed PCR-assay using a Qiagen TaqPolymerase assay (Qiagen, Hilden, Germany).

Core primer sequences for the three amplicons were as follows:

- 16S Primer fw: ACCGCGGCTGCTGGCAC
- 16S Primer rev: AGAGTTTGATCMTGGCTCAG
- ITS Primer fw: GTCCCTGCCCTTTGTACAC
- ITS Primer rev: CCTGGTTAGTTTCTTTTCCTCCGC
- 18S Primer fw: AACCTGGTTGATCCTGCCAGT
- 18S Primer rev: CTACGAGCTTTTTAACTGCAACAACTTTAATATACGC

To the core primer sequences, different 8 bp long internal barcode sequences were added, resulting in a dual barcoded amplicon sequencing approach.

Amplification was performed by preincubation at 98 °C for 5 min, 30 cycles of denaturation (94 °C for 30 s), annealing (cycle 1–5: 56 °C; cycle 6–30:65 °C for 30 s each) and elongation (72 °C for 60 s) and a final step of 72 °C for 10 min.

PCR products were purified with Ampure Beads, normalized via Sequelprep Plates (Thermo Fisher Scientific, Waltham, MA, USA) and pools of PCR products were ligated to Illumina compatible adapter sequences with a NEB DNA LibraryPrep-Kit (all according to the manufacturer’s recommendations). Sequencing was performed on a MiSeq instrument using Illumina MiSeq Reagent Kit v3 600-cycle MS-102-3003 with read1 and read2 reading length of 300 bases (Illumina, San Diego, CA, USA).

### 2.7. Analysis of Microbiome Sequencing Data

Self-Organizing Map (SOM) is a neural network algorithm for clustering complex data that successfully has been adopted for various microbial analyses [36,37,38]. Here, for each sample and dual barcoded primer set, sequences of 200 nt including primers were clustered by SOM algorithm [39] with 10,000 rounds of training in a two-dimensional 20 × 20 network using Hamming distance for different nucleotides. After mapping, each cluster was checked for average association between sequences by Pearson correlation. Clusters with R < 0.95 were subjected to subclustering in a 10 × 10 network. The best representative sequence closest to cluster average was submitted to BlastN homology search using genbank release gb240. Clusters were selected if alignment length was ≥200. The cluster size (number of sequences) was associated with the sequence identified by BlastN. An average of 215 different species per sample were identified. For each species, the frequency per sample was calculated by the ratio between the count of sequences per species and the count of all sequences selected from one sample.

### 2.8. Calculation of Pathway Frequencies Using the MACADAM Pathway Scores

For each bacterial species, pathways and corresponding pathway scores were selected from the MACADAM database where possible [40]. For each species, pathway scores were multiplied with the frequency of the species; all frequencies of the same pathway were added up to determine the overall score per pathway and sample.

### 2.9. Statistical Analysis

Statistical comparisons were performed using one-sided paired *t*-test for kinetics or Welch test for comparisons between RA and MetS. For statistical assessment of changes in the number of tender or swollen joints, the non-parametric Wilcoxon signed-rank test for paired samples was used.

## 3. Results

### 3.1. Study Population

We investigated patients with RA (*n* = 20) in comparison to MetS without concomitant inflammatory disease (*n* = 10). Both groups were comparable with respect to age (mean/min/max RA: 51/22/77; MetS: 59/29/71; *p* > 0.14) and gender (w/m RA: 19/1 MetS: 8/2; *p* > 0.25). Seropositive RA patients (*n* = 14) had RF (*n* = 12) and/or ACPA (*n* = 12). The body mass index (BMI) differed significantly (BMI_MetS_ = 35.1; BMI_RA_ = 25.1; *p* < 0.004). Ongoing immunosuppressive therapy in RA was accepted without restriction and kept stable. RA patients expected the treatment to improve their disease and/or reduce their medication subsequently. We enrolled independently of activity criteria and demonstrated that microbiota reduction had no negative effects even in patients with very low disease activity. The study was performed in an inpatient setting and was well tolerated, without premature discontinuation. After bowel cleansing and until breaking the fast, bowel movement was absent.

### 3.2. Microbiota Reduction Rapidly Reduced Disease Activity in Almost All RA Patients

Patients were monitored before intervention (T0), after bowel cleansing early during fasting (T1), at the end of fasting (T2), and 3 days after breaking the fast (T3) (Figure 1). Microbiota clearance was optimized with colonoscopy fluid according to the split-dose concept [30]. Subsequent fasting (<250 kcal/day, vegetable juices) with sugar-free tea or water ad libitum (≥2 L/day) already significantly reduced disease activity in RA patients within 3 days (*p* < 0.01). Clinical improvement (DAS28/SDAI; Table 1) increased over time and was most pronounced at the end of the study (T3 = 3 days after breaking the fast; *p* < 0.00001). This confirmed the assumptions made for calculating the necessary group size.

According to EULAR criteria [32], eight patients showed “good” (DAS28 change min/mean/max: −1.09/−1.76/−2.90), eight “moderate” (−0.65/−1.20/−1.59), three “non-response” (1.67/0.15/−0.87) and one patient with initial remission (DAS28 < 2.6) remained stable (−0.14). RA patients in remission increased from two (T0) to eight (T3). Boolean remission changed from none (T0) to three patients (T3). We observed this exceptional and rapid clinical response in both seropositive and seronegative RA patients and unrelated (R ≤ ±0.1) to age, disease duration, concomitant glucocorticoid treatment, conventional DMARDs or biologics, or BMI (Table 1). Individual DAS28/SDAI parameters for all patients confirmed this homogeneous improvement (Figure S2).

### 3.3. Non-Classical Monocytes Are the Most Sensitive and Specific Subset Indicating Response in RA

**Figure 2:**
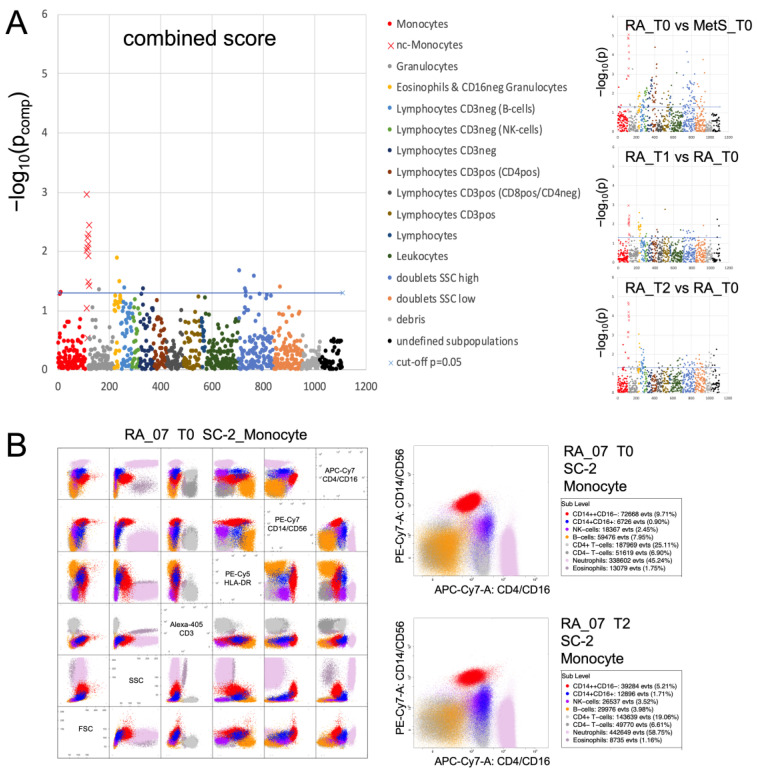
Cytometric profiling summary. FACS analysis of blood leukocytes was performed with 10 different staining cocktails [34] as indicated in the methods section. The immunoClust algorithm [35] identified about 110 different populations per staining cocktail. (**A**) All populations were compared by one-tailed paired *t*-test in RA between T0 and T1 or T2 and by Welch test between RA and MetS at T0. Test-results for all different populations (indicated by number on the abscissa) are presented on the ordinate as −log10(*p*) values with significance above the dashed line (−log_10_(0.05) ≈ 1.3). Monocytes and their subpopulations were detected in all 10 cocktails, albeit best with cocktails containing CD14 and CD16 staining and with varying degrees of separability in others. In all three group comparisons and also in the different staining cocktails that separated sufficiently the monocyte subgroups, non-classical monocytes revealed the dominant differences. This can be considered as a technical validation. The combined score (weakest *p*-value from all three comparisons) highlights this dominance when compared to all other populations. Significance refers to a decrease in RA_T0 compared to MetS_T0 and a gradual increase in RA_T1 and RA_T2 compared to RA_T0. (**B**) Dot plots of leukocyte populations are exemplarily presented for staining cocktail 2 (SC-2) in RA_07 with selected markers to indicate the separation of the relevant populations. Details of population characterization are summarized in Figure S1. The two plots with CD14/CD16 staining present the populations of RA_07 at T0 and T2 with an increase in non-classical monocytes at the end of fasting (blue).

**Figure 3:**
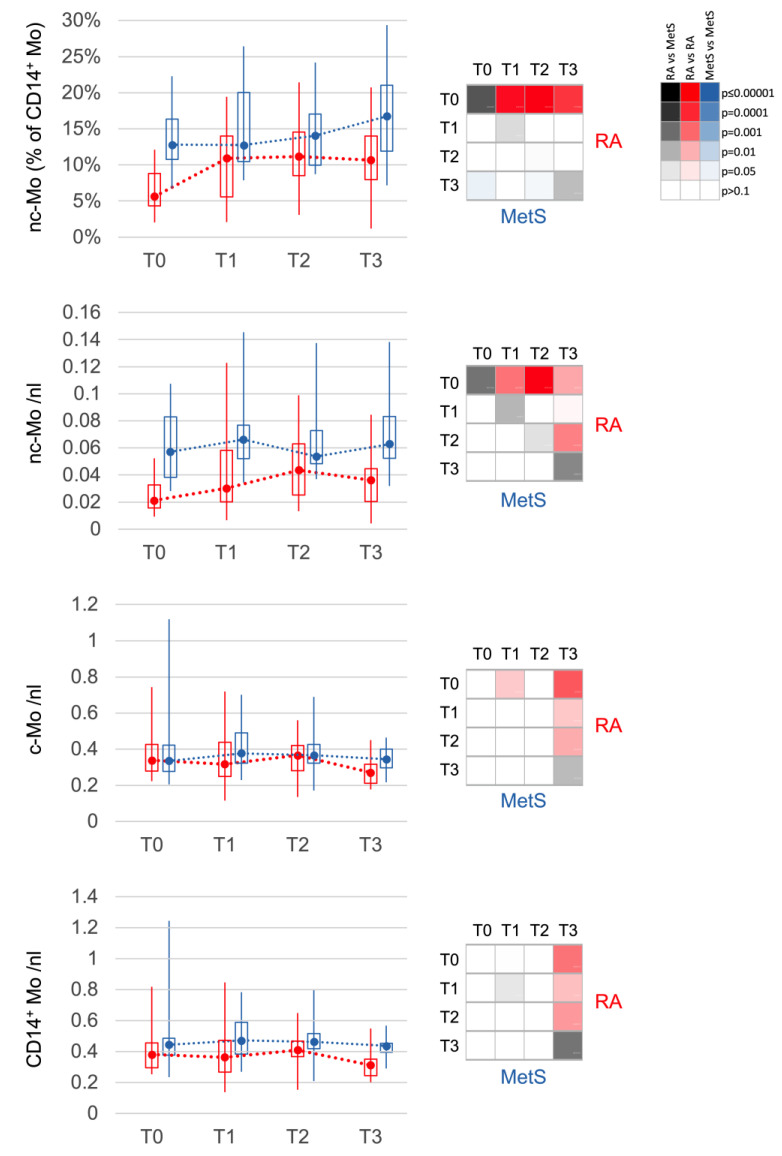
Changes in monocyte populations. Non-classical monocytes (nc-Mo) are decreased at T0 in RA (red) when compared to MetS (blue). They increase after bowel cleansing and during fasting in RA until T2. After fast breaking, monocytes, classical (c-Mo) and nc-Mo, decrease slightly but significantly in RA between T2 and T3. The distribution of values is displayed in boxplots (upper and lower quartile) with whiskers (maximum and minimum) and median. The 4 × 4 panel displays the significance of the corresponding comparisons between the different time points T0 to T3 for RA (red, top right) and for MetS (blue, bottom left), as well as between RA and MetS at the same time points (grey, diagonal). The color intensities corresponding to the significance level are shown in the 3 × 6 panel. Values are calculated in % of all monocytes or cells per nanoliter (nL) of blood.

Routine differential blood counts detected only effects common to both RA and MetS with a reduction in total leukocyte count after 10 days and a decrease in eosinophils and lymphocytes already at T1 (*p* < 0.05). In contrast, cytometric profiling with 46 different surface markers in ten different staining cocktails identified that among all leukocyte populations, changes in RA non-classical (CD14^+^CD16^+^) monocytes were dominant (Figure 2). At baseline, this monocyte phenotype, which slowly develops in the blood [41,42], was reduced in absolute and relative quantity in RA compared to MetS. It increased upon microbiota reduction towards levels of MetS/controls (Figure 3; Figure S3) while the phenotype released from the bone marrow [26,42], the classical monocytes (CD14^++^CD16^-^), declined within the first few days more than the total monocyte count (Figure 3). In patients with MetS, classical and non-classical monocytes did not change significantly. Interestingly, of the three non-responders according to DAS28 response criteria, (i) RA_13 showed a constant deterioration in DAS28, with a concomitant decrease in non-classical monocytes and in contrast to others without ketone bodies in urine upon microbiota reduction, suggesting inadequate carbohydrate restriction; (ii) RA_02 had the highest initial disease activity, but responded insufficiently according to EULAR criteria (DAS28_T0_ = 7.05; DAS28_T3_ = 6.18) and showed an increase in absolute but not relative non-classical monocyte counts; (iii) RA_14 received the highest prednisolone treatment, had the lowest absolute and relative non-classical monocyte counts and deteriorated at T1 before returning to baseline levels.

**Figure 4:**
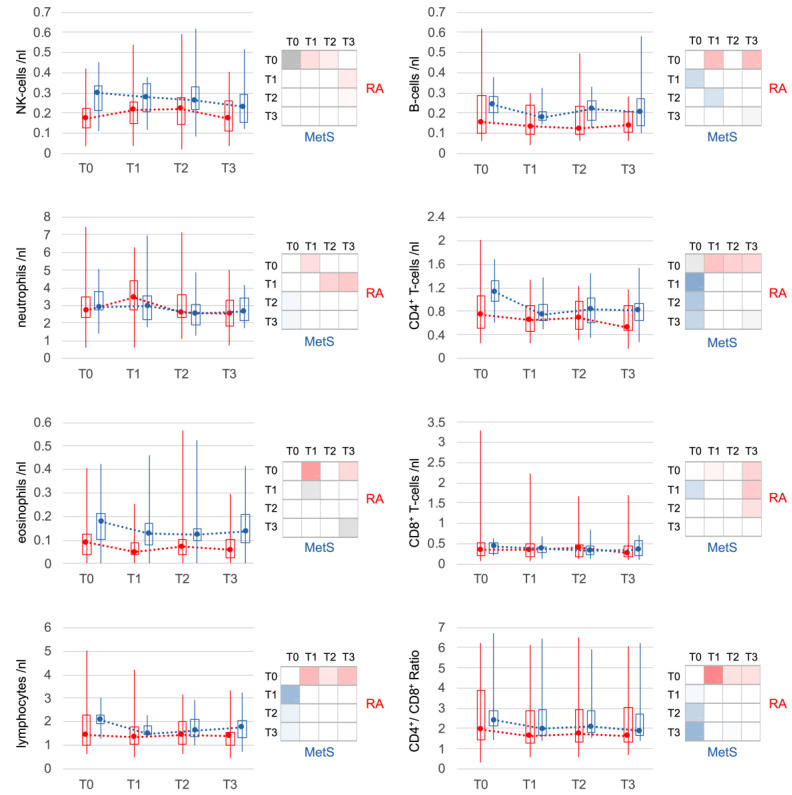
Changes in other major leukocyte populations. Less pronounced but significant changes were also seen for other leukocyte populations. In RA, changes in NK cells were weaker but comparable to those of non-classical monocytes. Lymphocytes as a whole and the subpopulations of T-cells and B-cells showed similar changes in RA and MetS patients. Only neutrophils and eosinophils showed slight differences. Neutrophils increased transiently (T1) only in RA and eosinophils changed, although similar, with significance only in RA. Statistical significances of comparisons were indicated as described in Figure 3.

RA blood counts of NK cells, which were slightly reduced at baseline, increased after microbiota reduction. Neutrophils also increased significantly in the first 3 days, but returned to baseline at T2. In MetS, both NK cell and neutrophil counts remained stable on average. Lymphocytes, especially CD4^+^ T cells and B cells, declined significantly in both RA and MetS within the first 3 days. CD8^+^ T cells contributed less, resulting in a significant decrease in the CD4^+^/CD8^+^ T-cell ratio in both groups. The decline of eosinophils in the first 3 days in both groups reached significance only in RA (Figure 4).

### 3.4. Serum Inflammatory Markers Declined upon Microbiota Reduction

**Figure 5:**
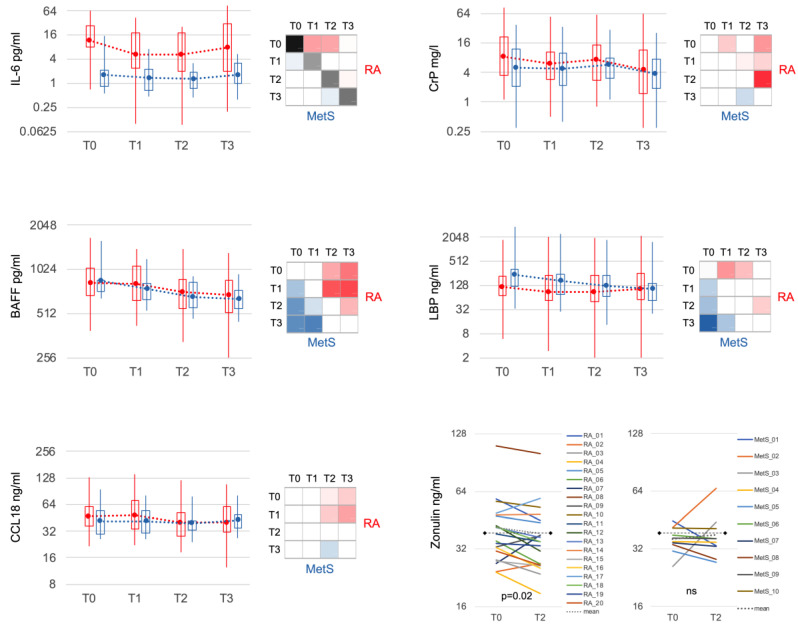
Selected serum markers of immune response and mucosal barrier. IL-6 was significantly increased in RA compared to MetS at all time points and showed markedly significant reduction in RA after bowel cleansing and during fasting. CCL18 was slightly but not significantly increased in RA compared to MetS and, in contrast to MetS, significantly decreased during the course (T2 and T3). CrP decreased significantly in RA initially (T1) and especially after fasting (T3). BAFF and LBP behaved similarly in RA and MetS. Zonulin showed significantly higher variance in RA and was more often above the threshold for normal values (38 ng/mL; dashed black line) at baseline. Bowel cleansing and fasting led to a significant decrease only in RA. IL-6: interleukin 6; BAFF: B cell activating factor; CrP: C-reactive protein; LBP: lipopolysaccharide binding protein.

IL-6 demonstrated the most significant difference between RA and MetS at all time points (Figure 5). The elevated levels in RA at baseline decreased within 3 days at T1. B cell activating factor (BAFF) dropped significantly in both groups with comparable mean values but greater variation in RA. CC-chemokine ligand 18 (CCL18), a cytokine of innate immune cells, diminished from initially slightly higher values in RA to levels comparable to MetS at T2. CrP, also moderately elevated in RA at baseline, decreased significantly at T1, but rebounded towards the end of the fast. Lipopolysaccharide binding protein (LBP), another acute phase protein, was slightly lower in RA compared to MetS at T0 and decreased in both groups during fasting. Zonulin, a marker of mucosal permeability, was more frequently elevated in RA at T0 (>38 ng/mL: RA *n* = 9; MetS *n* = 3) and significantly declined only in RA (mean_T0_ = 41.6 ng/mL; mean_T2_ = 38.6 ng/mL; *p* < 0.05) but not MetS (mean_T0_ = 36.2 ng/mL; mean_T2_ = 38.0 ng/mL).

### 3.5. Effects of Bowel Cleansing with Fasting on Metabolites and Cortisol

**Figure 6:**
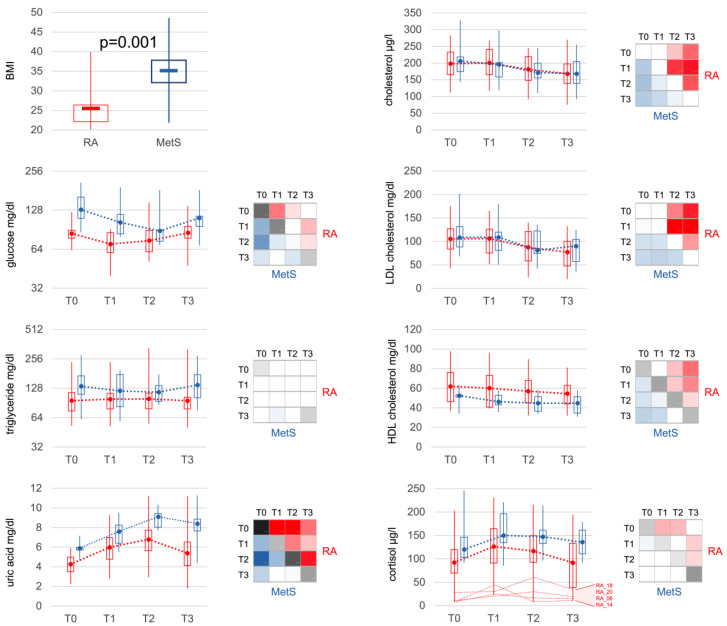
Metabolic markers. Besides a significant difference in BMI, metabolic markers at baseline showed significantly increased levels of glucose, triglycerides, uric acid, HDL-cholesterol and also cortisol in MetS patients. Bowel cleansing and fasting led to comparable changes in RA and MetS. Interestingly, the difference for HDL-cholesterol remained significant between RA and MetS at all time points. Cortisol increased in both groups, was lower in RA than in MetS at all time points and only showed a slightly more significant increase in the early phase (T1), which can be explained by the very low levels in some patients with apparent partial adrenocortical insufficiency. Statistical significances of comparisons were indicated as described in Figure 3. BMI: body mass index; LDL: low-density lipoprotein; HDL: high-density lipoprotein.

Corresponding to BMI, glucose, triglycerides and uric acid were significantly elevated in MetS at baseline. Bowel cleansing with fasting led to a decrease in glucose and an increase in uric acid similarly in both groups (Figure 6). Triglycerides remained unchanged in RA patients but declined in the majority of MetS patients. Cholesterol (total, LDL, HDL) also decreased in both groups, with HDL levels consistently lower in MetS than RA. Cortisol levels rose in both RA and MetS, being significantly higher in MetS compared to RA at all time points.

### 3.6. 16.S rRNA Sequencing of the Gut Microbiota Indicated a Shift towards “Generalist Species” after Fasting

At baseline, few differences were found between gut microbiota in RA (*n* = 15) and MetS (*n* = 7), which concentrated on the phylum *Actinobacteria* and at the family, genus and species level on the phylum *Firmicutes* (Table S2A), especially species of the order *Clostridiales*. *Ruminococcus albus* decreased in both groups and was the only species that was more abundant in RA compared to MetS at T0 (*p* = 0.029) and T3 (*p* = 0.045; Table S2A,B).

Filtering for RA-related microbes at T0, which were absent in MetS at T0 and T3, identified 166 species. Of these, 135 were found in different patients but each species in only one patient, 23 in two, 7 in three and 1 in six patients with RA. Although detectable at very low frequency (<1 out of 10^3^ microbes), this included nine species reportedly found in septic arthritis (nine patients before and six after fasting) and seven species associated with RA (eight patients before and four after fasting; Table S3). No bacterial species were found in all RA patients but not MetS (Table S2E). Very few differences between RA and MetS showed a correlation with BMI (R > 0.4/< −0.4; Table S2A,B,E).

**Figure 7:**
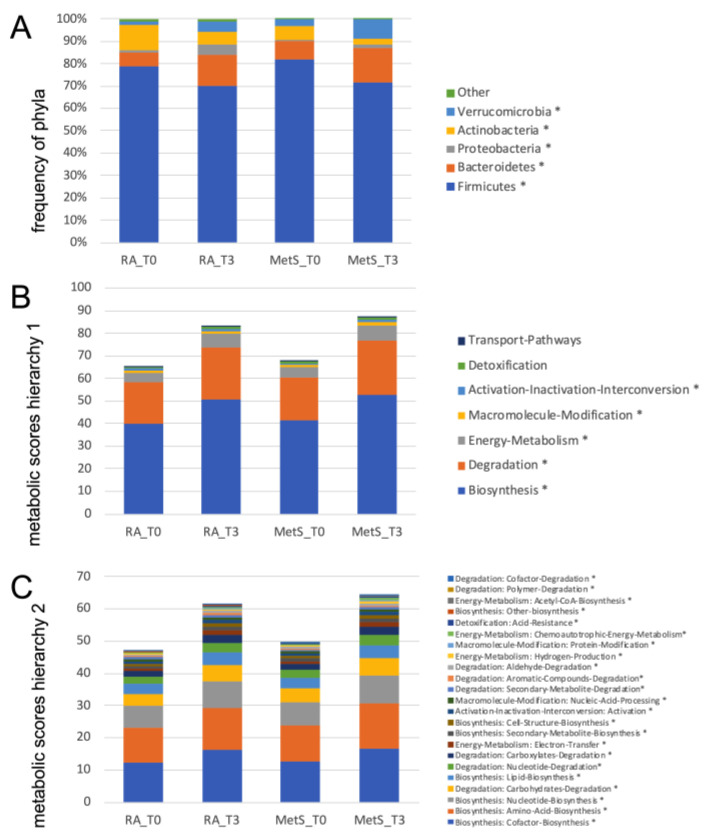
Frequencies of gut microbiome phyla and metabolic scores. (**A**) This graph summarizes the mean abundances of phyla before (T0) and after fasting (T3) in RA and MetS patients. For *Firmicutes*, *Bacteriodes*, *Proteobacteria*, *Actinobacteria* and *Verrucomicrobia* (*Akkermansia muciniphila*), the changes are similar in RA and MetS patients. (**B**,**C**) Metabolic activities were scored based on annotations in the MACADAM database by multiplying the abundance of each species in each sample with each associated metabolic score in the MACADAM database. Scores were summed separately for each pathway in each sample and then for each higher-level annotation hierarchy. Mean scores for annotation hierarchy 1 (**B**) and 2 (**C**) were calculated and presented for RA and MetS before and after fasting. They show a mean increase for (**B**) biosynthesis, degradation, and energy metabolism and in detail (**C**) the increase in synthesis of co-factors, amino acids, nucleotides, and lipids but also degradation of carbohydrates in the same way in RA and MetS after fasting. * *p* < 0.05 for comparisons between T0 and T3.

**Figure 8:**
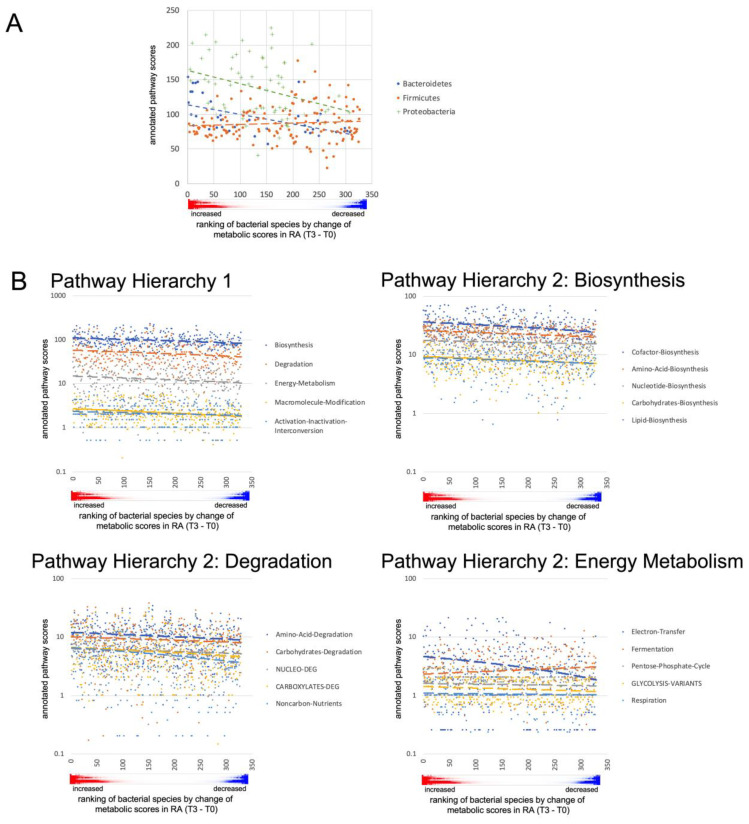
Pathway scores of changing microbial species. (**A**) For each bacterium in the phyla *Bacteroidetes*, *Firmicutes* and *Proteobacteria* that changed in RA patients between T0 and T3, the scores annotated in the MACADAM database for the pathways of biosynthesis (H1 category biosynthesis) were summed. Bacteria were sorted on the abscissa according to the mean increase in metabolic activity after fasting, (the red/blue color plot of the mean values from Table S5A is shown below the abscissa in (**A**,**B**)). *Bacteroidetes* and *Proteobacteria* that increased after fasting were annotated in MACADAM with higher scores for biosynthesis compared to those that decreased. Species of the phylum *Firmicutes*, which increased after fasting had lower metabolic activity compared to those that decreased. However, there was an overall decrease in *Firmicutes* compared to an increase in *Bacteroidetes* species after fasting. (**B**) The sum of pathway scores annotated in the MACADAM database from the categories of H1 was higher in species that increased on average after fasting than in species that decreased. For several dominant categories of hierarchy 2, belonging to biosynthesis, degradation and energy metabolism, these differences were also evident at the level of the annotated scores.

Fasting resulted in much more pronounced differences. In RA patients, *Actinobacteria* and *Firmicutes* decreased significantly, while *Bacteroidetes* and *Verrucomicrobia* (*Akkermansia muciniphila*) increased (Figure 7A, Table S2C,D). Further taxonomic sorting down to the species level displayed individual changes rather than general effects of fasting. Assuming that differences in metabolic capabilities between higher order of grouping than species contribute to this observation, we calculated virtual metabolic scores for each sample based on species abundance and species-specific annotation of pathway scores in the MACADAM database [40]. This approach suggests that metabolic capabilities increase after fasting, particularly in the areas of biosynthesis, degradation, and energy metabolism (Figure 7B,C, Table S4). The calculated metabolic switch matched the observed changes in microbial abundances at phylum level: the species of the phylum *Bacteroidetes*, that increased after fasting, were annotated with higher pathway scores, as were many of the proteobacteria that also increased. In contrast, the significantly reduced *Firmicutes* species were assigned fewer metabolic capabilities (Figure 8). Thus, after fasting, a shift towards a category of species with a broader metabolic repertoire relevant to microbiota expansion was observed (Table S5A,B).

### 3.7. Internal Transcribed Spacer (ITS) and 18S rRNA Sequencing Indicate a Higher Prevalence of Eukaryotic Microbes in RA

Eukaryotic species occurred in significantly lower numbers than procaryotic species in both RA and MetS (mean number of species per RA/MetS sample: ITS: 12.0/8.7; 18S: 5.4/6.8; 16S: 226.2/228.0) with correspondingly decreased Simpson’s index of diversity (probability of two randomly selected sequences of a sample belonging to different species; ITS:0.48/0.36; 18S:0.39/0.41; 16S:0.94/0.92). Group comparisons similar to 16S rRNA data suggested some differences for eukaryotic microbes (*p* < 0.1), which almost all dominated in RA (including *Candida glabrata*, *Colpodella angusta*, *Blastocystis species*, *Kazachstania taianensis*, *Pichia membranifaciens*, *Hanseniaspora uvaru*m, *Alternaria alternata*, *Cladosporium cladosporioides*; Table S6A,B). No eukaryotic colonizer was present in all and only RA patients. The increased presence in RA became more apparent when combining results of T0 and T3 for each patient and was clearly significant (*p* < 0.001), when eukaryotic frequencies were added per donor for species with difference between RA and MetS (Table S6C).

## 4. Discussion

This study demonstrated that extensive gut microbiota reduction by bowel cleansing and fasting for at least one week can rapidly reduce clinical and inflammatory activity in RA. The dominant change was the decrease in the elevated monocyte turnover, which is also a dominant process of RA immunopathology. In addition, serum IL-6 and zonulin, a marker of mucosal barrier dysfunction declined. Changes in the gut microbiome after fasting suggested a reduction in bacterial dysbiosis in RA. Moreover, eukaryotic colonizers were found more frequently in RA than in MetS, suggesting possible direct or indirect involvement in RA.

The most important finding in RA compared to MetS was the stepwise increase in non-classical monocytes after microbiota reduction. Non-classical monocytes differentiate from classical monocytes over several days during circulation in the blood [41,42]. Inflammatory diseases that attract monocytes from the blood into the inflamed tissue shorten their circulation time. Consequently, fewer non-classical monocytes can develop. Recently, we described this phenomenon as a marker for increased monocyte turnover in RA by demonstrating increased production in the bone marrow, premature release into the bloodstream, and reduced differentiation to the non-classical phenotype [26]. In this study, we confirmed the reduced level of non-classical monocytes and thus increased monocyte turnover in RA patients at baseline. This finding was present at baseline even in RA patients under targeted immunosuppression with low clinical disease activity.

Recently, we demonstrated that monocytes also play an important role in RA synovitis by showing that tissue transcriptomes are dominated by activated monocytes/macrophages [27]. Many monocyte-derived cytokines, chemokines and proteinases are increased in the synovial fluid more than in the peripheral blood of RA patients and synovial tissue transcriptomes of RA compared to osteoarthritis patients matched closely to transcriptomes of monocytes stimulated with bacterial or fungal components rather than viruses, specific cytokines or immune complexes [27]. These exceptional activation patterns of monocytes/macrophages confirm and extend the recently reported functional diversification of macrophages in RA synovium identified by single cell sequencing [43]. The increased levels of monocyte cytokines, chemokines and proteases in the joint compared to the blood support the hypothesis that the signature of monocytes originates directly in the joint and consequently that microbial factors or mimicking antigens appear to be present in the joint.

**Figure 9:**
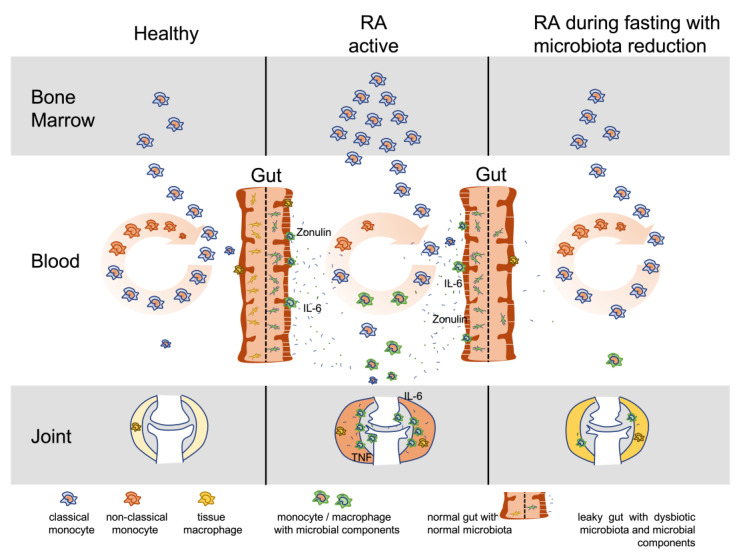
Model for microbiota involvement and monocyte turnover in RA. This model illustrates the turnover of monocytes in active RA compared to healthy and RA during fasting with microbiota reduction. Dysbiotic gut microbiota induces inflammation, leaky gut and spreading of microbial components into the body with affinity to joint tissue structures, especially components of the extracellular matrix. Monocytes become involved with accumulation and activation in the gut and inflamed tissue sites. Increased monocyte involvement induces increased production in the bone marrow and increased migration from the blood circulation into the inflamed tissues. Shorter circulation time reduces maturation in the blood from classical (released from bone marrow) to non-classical monocytes, which represent the phenotype with reduced capacity for activation by lipopolysaccharides (decreased CD14 expression) or migration into tissue (decreased L-selectin expression) but increased capacity for clearing of immune complexes (elevated CD16 = FcγRIII expression). Microbiota reduction and fasting in RA reduces microbial triggers and thus monocyte turnover, joint inflammation, and levels of IL-6 and zonulin. IL-6: interleukin 6; TNF: tumor necrosis factor.

Against this background, intestinal cleansing and fasting to reduce the largest microbial colonization of the body gains particular importance. Our results, indicating that virtually all RA patients experienced clinical improvement and an increase in the non-classical monocyte population within a few days, together with a decrease in serum inflammatory markers, can be taken as an indirect sign that disease activity in RA is maintained by components of the intestinal microbiota (Figure 9).

This hypothesis is further supported by the kinetics of zonulin, which suggests that the disturbed mucosal barrier improved after microbiota reduction. Zonulin, also known as pre-haptoglobin 2, a human protein analogue to the Vibrio cholerae derived zonula occludens toxin, modulates intestinal permeability and is found increased in intestinal tissues under inflammatory conditions like coeliac disease [44] but also in stool and serum/plasma samples of inflammatory bowel disease (IBD) when compared to healthy controls [45]. Along with measurements of transepithelial resistance, endotoxins and lipopolysaccharide, the decrease in serum zonulin was confirmed in a meta-analysis of 26 randomized controlled trials as an indicator of improved intestinal barrier function when probiotics are administered [45]. Targeting of zonulin with larazotide attenuated collagen induced arthritis (CIA) in mice [22]. Zonulin also seems to indicate and may even contribute to a disturbed intestinal barrier in human arthritis [46]. However, the increase in zonulin in RA seems to depend on microbial stimuli, as it decreases after microbiota reduction. It may be pharmacologically blocked in CIA but this does not completely prevent arthritis. In IBD, barrier dysfunction seems to improve with probiotics, which most likely act by competing with and reducing pathogenic microbes. While suggested that loss of mucosal barrier may be an important contributing factor to autoimmunity including rheumatoid arthritis [47], these previous findings do not exclude that constant triggers of pathogenic microbes do not only maintain barrier dysfunction but may also directly contribute to the pathogenesis of RA with defined microbial products spreading into the human body. Not all individuals with increased zonulin levels and barrier dysfunction have arthritis. Particular microbial factors seem to be necessary. The similarity of inflammatory transcriptome patterns of RA synovitis tissue with microbial triggering of monocytes suggests that microbial products or mimicking antigens need to act at the synovial tissue site. In fact, bacterial peptidoglycans have been described in the joints of RA and other arthritides [48].

The improvement in RA treatment with the new targeted therapies also supports the importance of monocytes in disease activity by either i) blocking monocyte-derived cytokines (TNF, IL-6), ii) suppressing monocyte activation by T cells via CTLA4 (abatacept), or iii) inhibiting Janus kinases in intracellular signaling following cytokine stimulation, particularly by TNF and IL-6 [49]. Interestingly, increased monocyte turnover also improved in RA patients who had started under the control of targeted therapies with low disease activity or remission. This suggests that targeted therapies can suppress monocyte activation but not sufficiently monocyte recruitment, while extensive microbiota reduction attenuated both monocyte recruitment/turnover and activation (IL-6 production) in RA.

In principle, indirect mechanisms are also conceivable. For example, processes like vasoconstriction, production of reactive oxygen species and hypoxia can support inflammation and, if induced by the gut microbiota, would decrease upon microbiota reduction [50,51]. On the other hand, metabolic changes during fasting such as the production of ketone bodies may act in an immunosuppressive manner [52]. However, these effects are general in nature and, if strong enough and relevant, should also be observed in the MetS control group. In a similar way, the increase in endogenous cortisol production during fasting was considered to contribute to clinical improvement [25]. However, when recent data on cortisol half-life in vivo [53] are applied for calculation of the increased production during fasting, this results in not more than 2.5 mg of prednisolone equivalent per day (Table S7). This amount is obviously insufficient to explain the observed decline in disease activity. A contribution can also be excluded especially in RA patients with low endogenous cortisol levels and minor increase during fasting, which was probably due to partial adrenocortical insufficiency after glucocorticoid therapy [54]. Cortisol levels correlated negatively with blood glucose levels, and MetS patients achieved higher levels and changes than RA patients, without effects on the immune markers that changed in RA. Thus, the observed increase in endogenous cortisol may indicate a stress response following hypoglycemia sufficient to stabilize fasting metabolism but insufficient to produce relevant therapeutic effects on inflammation.

If the gut microbiome of RA patients is the key trigger of disease activity and a pathogenic component can be lowered quantitatively by fasting, inflammation should return at a lower level upon breaking the fast and microbiome re-expansion. Indeed, we found a decrease in total monocyte count, including both, classical and non-classical monocytes, and an increase in IL-6 between T2 and T3 (*p* < 0.1), albeit much less when compared to baseline levels.

Besides a decrease in monocyte turnover upon microbiota reduction in RA, a decrease in neutrophil turnover seems to occur as well. Neutrophils are the leading population in the synovial fluid of inflamed RA joints [55]. The sudden reduction in microbial triggers along with reduced consumption and an obviously delayed decline in granulocyte production in the bone marrow resulted in an accumulation of neutrophils in the blood during the first three days of fasting. Conversely, the slowly decreasing bone marrow activity until breaking the fast could not immediately compensate for the subsequently increasing demand of neutrophils, so that the blood levels declined.

After breaking the fast, clinical activity and immune parameters remained improved, and as shown recently, were even stable for at least three months when plant-based or guideline-based anti-inflammatory diets were applied [15]. Kjeldsen-Kragh illustrated that stabilization at lower activity levels is achievable even for up to one year when individually optimized diets are applied [13].

These observations could be explained by our 16S sequencing results. After fasting, the re-expanding microbiome is obviously shaped by the changing demand for metabolic capabilities. Bacterial cell division and expansion depend on increased energy supply and, in particular, on the formation of nucleic acids, amino acids, lipids and co-factors of biosynthesis. Indigestible food remnants that enter the colon after enzymatic degradation and absorption in the small intestine seem to allow the colonization of primarily those microbial species that fulfil the required enhanced metabolic activities and withstand patient-specific selection factors such as specific immune responses or genetically determined barrier functions of the mucosa [56,57,58,59]. Compared to the metabolic changes in microbiome starving on very low-calorie diets [16], our study shows quite opposite metabolic properties of the microbiome when expanding after fasting, which may play a pivotal role for the subsequent outcome. Very low-calorie diets may create the basis for such changes by the quantitative reduction in the microbiota. For expansion, however, the gut microbes require extensive metabolic capabilities for re-growth from food components indigestible in the human small intestine. These microbes appear to correspond to a so-called generalist phenotype. Considering this in terms of evolutionary mechanisms, it can be assumed that this phenotype belongs to the gut-adapted species that have selected barrier functions and tolerance as protective mechanisms in the host gut and are therefore less harmful and less pathogenic. Fasting could open a window for subsequent dietary interventions to support this selection process and thereby reduce or even displace pathogenic germs. This hypothesis is supported not only by the successful dietary strategies of Kjeldsen-Kragh [13], but also by the principle of natural defense against intestinal infections through diarrhea reaction and loss of appetite. To support the expansion of favorable microbes after fasting, probiotics [60] or supplements such as polyphenols [61,62] with proven beneficial influence on RA could be useful. This would need to be tested in appropriate follow-up studies. In general, a better understanding of the interactions between food and the microbiome is needed to maintain or even improve the reduction in inflammation achieved by fasting with appropriate diet and nutrition.

Typing of eukaryotic species suggested additional and possibly more permanent dysbiotic problems in RA patients. Although with lower detection rate and diversity, fungal colonizers in particular occurred more frequently in RA, and some were identified repeatedly in the same donor. Given the lower detectability and the associated difficulty in obtaining convincing statistical results, these data can currently only stimulate more sophisticated analyses of the involvement of eukaryotes in RA pathogenesis. Eukaryotic colonizers, especially fungi, are much more adaptable than bacteria, and probably have more strategies for persistence and pathogenicity. Cell walls of fungi may contain large amounts of citrulline [63], which might be linked to the increased occurrence of ACPA in RA. Additionally, immunological defense mechanisms against fungi include NK cells [64,65], which in our study showed strikingly similar kinetics to non-classical monocytes.

With these indications that the gut microbiota may drive the inflammatory process in RA, the question arises as to how the previously considered multifactorial causes of RA consisting of genetic, hormonal, and environmental factors can be classified into the concept proposed in Figure 9. This requires a very precise analysis of the gut microbiota in patients who respond sustainably to dietary changes, in order to identify possible etiologically involved microbes in a more targeted manner and thus to perform further functional investigations on the development of arthritis.

## 5. Conclusions

In summary, extensive reduction in the gut microbiota in RA shows a strong and consistent effect on clinical improvement, with a comparable strong and consistent effect on reducing the increased monocyte turnover in RA. The high significance of the clinical improvement confirmed the assumptions made for the calculation of the group size. The dominant immunological effect could also be identified with this group size, as it occurred with a comparably strong effect.

Since increased monocyte turnover is also a leading immunologic process in RA and the transcriptional patterns of RA synovitis are most consistent with microbial stimulation of monocytes, our observations confirmed the initial hypothesis and suggest that a major inflammatory stimulus in RA originates from the gut microbiota.

Regarding the study of the microbiome, the group size was clearly too small. Only the shift after fasting to species with increased metabolic capabilities, which occurred with a strong effect in both, RA and MetS, was highly significant and seems to reflect a general mechanism that may also be helpful in other diseases. However, to identify relevant microbial species, larger patient groups and deeper sequencing would be required. It is also likely that different microbial species may be involved in different RA patients. To better answer this question, follow-up studies of the gut microbiota over a longer period of at least three months after fasting would be needed, together with testing of suitable diets.

The usually sterile inflammation in RA joints with a monocyte transcriptional imprint comparable to microbial stimulation suggests that microbial products cross a deranged mucosal barrier or mimicking antigens represent a relevant trigger [22,23,28]. Other immunological features previously associated with autoimmunity, like RF and ACPA, were not affected by fasting. Bacteria may be of relevance as suggested by (i) their dominance in the gut, (ii) previous findings of bacterial peptidoglycans in the joint [48], and (iii) the synovitis transcriptome patterns best fitting to monocytes triggered by bacterial components [27]. Nevertheless, dysfunction of the mucosal barrier may also involve eukaryotic microbes like fungi, which have been found to be increased in RA, may provide a pathogenic link to ACPA and may act supportively, if not essentially, in this disease.

## Figures and Tables

**Table 1:** Summary of clinical data. Patients are grouped according to treatment characteristics as indicated on the left side. All groups include seropositive and seronegative patients of different ages and disease duration. All except for one patient were female. DAS28 and SDAI values are colored for each patient from highest (red) to lowest (blue). DAS28 response is indicated according to EULAR criteria [32] and remission indicated when DAS28 was below 2.6 at T3. SDAI response is given in percent reduction and was minor (MiR; >50% and <70%), moderate (MoR; >70% and <85%) or major (MaR; >85%) with remission when SDAI < 3.3. f: female; m: male; BMI: body mass index; RF: rheumatoid factor; ACPA: anti-citrullinated-peptide-antibodies; Pred: prednisolone; cDMARD: conventional disease modifying antirheumatic drug; bDMARD: biological DMARD; DAS28: disease activity score 28-joint count; SDAI: simplified disease activity index for RA; MTX: methotrexate; LEF: leflunomide; SSZ: sulfasalazine; HCQ: hydroxychloroquine; TOC: tocilizumab; ETN: etanercept; CZP: certolizumab-pegol; ABT: abatacept; ADA: adalimumab; RTX: rituximab; NR: DAS28 non-response; MR: DAS28 moderate response; GR: DAS28 good response; SR: DAS28 stable remission.

	Patient	Age	Gender	BMI	Diseased	RF	ACPA	Pred	Current Therapy		Previous Therapy		DAS28				DAS28 Response				DAS	SDAI				SDAI Response				SDAI
		(Years)			(Years)	(U/L)	(U/L)	(mg/d)	cDMARD	bDMARD	cDMARD	bDMARD	T0	T1	T2	T3	T1	T2	T3	Best	Remission	T0	T1	T2	T3	T1	T2	T3	Best	Remission
no immunosupressive medication	RA_01	49	f	32.4	6.9	59.3	184				MTX		4.06	4.08	4.10	3.17	NR	NR	MR	MR		11.3	11.8	9.6	7.3	4%	−15%	−35%		
	RA_03	54	f	27.2	0.3	14	0.9						4.46	4.02	3.18	2.50	NR	GR	GR	GR	Remission	15.1	12.9	10.0	7.5	−15%	−34%	−50%	MiR	
	RA_09	22	f	17.2	21.2	14	0.5						5.54	4.63	4.49	3.98	MR	MR	MR	MR		41.4	41.4	41.1	36.3	0%	−1%	−12%		
	RA_13	61	f	24.0	11.3	49.9	300				MTX	TOC	4.97	5.76	5.68	6.64	NR	NR	NR	NR		24.3	33.5	27.3	46.0	38%	12%	89%		
Prednisolone	RA_08	65	f	20.0	5.1	935.4	272	2			MTX		4.42	3.92	3.63	3.24	NR	MR	MR	MR		30.8	21.7	22.9	19.4	−30%	−26%	−37%		
	RA_16	41	f	25.4	0.9	14	0.4	2.5					2.32	2.18	2.47	2.18	SR	SR	SR	SR	Remission	6.3	4.6	5.7	4.9	−27%	−10%	−22%		
	RA_12	31	f	22.7	7.3	70.6	212	5					3.13	2.75	1.99	2.48	NR	MR	MR	MR	Remission	13.0	7.4	6.0	6.3	−43%	−54%	−52%	MiR	
	RA_18	65	f	20.5	4.6	14	340	5					4.98	4.47	3.98	3.17	NR	MR	GR	GR		26.3	22.2	18.1	14.8	−16%	−31%	−44%		
	RA_19	70	f	25.1	6.6	14	0.4	5			MTX		5.96	5.37	4.64	4.37	NR	MR	MR	MR		33.5	23.5	21.3	19.7	−30%	−36%	−41%		
cDMARD ± prednisolone	RA_05	77	m	20.6	36.1	31.3	67		MTX				5.04	3.23	2.66	2.58	MR	GR	GR	GR	Remission	19.5	8.8	12.2	5.6	−55%	−37%	−71%	MoR	
	RA_04	50	f	21.5	1.7	31.3	340	5	MTX				1.82	1.25	1.05	0.49	SR	MR	GR	GR	Remission	3.6	2.8	2.7	0.1	−22%	−25%	−97%	MaR	Remission
	RA_17	52	f	20.8	8.6	104	285	5	LEF		MTX		4.78	4.70	3.21	3.75	NR	MR	MR	MR		24.7	23.6	18.1	17.6	−4%	−27%	−29%		
	RA_11	72	f	27.8	16.1	195.1	275	6	MTX				5.20	5.03	4.45	3.96	NR	MR	MR	MR		28.4	20.8	14.0	10.5	−27%	−51%	−63%	MiR	
	RA_02	63	f	32.9	14.9	14	0.4	10	MTX			ABT RTX	7.05	6.26	6.70	6.18	NR	NR	NR	NR		49.9	36.8	44.9	41.2	−26%	−10%	−17%		
bDMARD ± prednisolone	RA_10	53	f	25.5	35.2	600	12.4			TOC	MTX, SSZ, HCQ	ADA ETN	2.64	1.93	1.41	1.55	MR	GR	MR	GR	Remission	11.1	7.6	3.2	2.6	−32%	−71%	−77%	MoR	Remission
	RA_07	52	f	39.9	0.9	151	60	5		ETN	MTX		5.83	4.57	4.31	2.93	MR	MR	GR	GR		38.3	24.4	21.2	7.1	−36%	−45%	−81%	MoR	
	RA_14	34	f	23.9	20.3	119.7	30	15		CZP			3.97	5.16	3.89	3.61	NR	NR	NR	NR		19.3	27.6	19.4	16.3	43%	1%	−16%		
bDMARD & MTX & prednisolone	RA_20	61	f	26.1	25.6	14	0.8	5	MTX	ABT			4.93	5.06	4.26	3.49	NR	MR	MR	MR		24.7	25.1	15.4	14.5	2%	−38%	−41%		
	RA_06	22	f	25.5	6.1	240.4	2.6	7.5	MTX	ADA	LEF	CZP TOC	3.24	2.62	1.93	1.96	MR	GR	GR	GR	Remission	11.4	6.0	5.5	1.7	−47%	−52%	−85%	MaR	Remission
	RA_15	30	f	22.3	1.9	14	136	10	MTX	ETN		ADA ABT	3.45	3.17	2.51	2.23	NR	MR	GR	GR	Remission	13.6	11.1	6.1	6.0	−18%	−55%	−56%	MiR	

## Data Availability

The data presented in this study are available on request from the corresponding author. 16S, 18S and ITS sequencing data are provided in the SRA database (PRJNA987148).

## Supplementary Materials

The following supporting information can be downloaded at: https://www.mdpi.com/article/10.3390/jcm12134359/s1, Figure S1: Annotation of meta-clusters. Figure S2: Improvement of disease activity. Figure S3: non-classical monocytes. Table S1: Cytometric staining matrix. Table S2: 16S sequencing. Table S3: species reported in RA. Table S4: bacterial pathways. Table S5: pathway changes and species. Table S6: 18S ITS sequencing. Table S7: endogenous cortisol induction. Legends: Legends Tables S1–S7.
