# Supplementary material for: Intestinal Microbiota Reduction Followed by Fasting Discloses Microbial Triggering of Inflammation in Rheumatoid Arthritis
Source: J Clin Med. 2023 Jun 28;12(13):4359. doi: 10.3390/jcm12134359 (PMC10342944; doi:10.3390/jcm12134359)

Figure S1

Annotation of meta-clusters

Populations were annotated according to the subsequent steps of population selection, corresponding to the strategy of conventional gating. The populations with their corresponding colour are indicated in the upper right corner of each plot.

all events

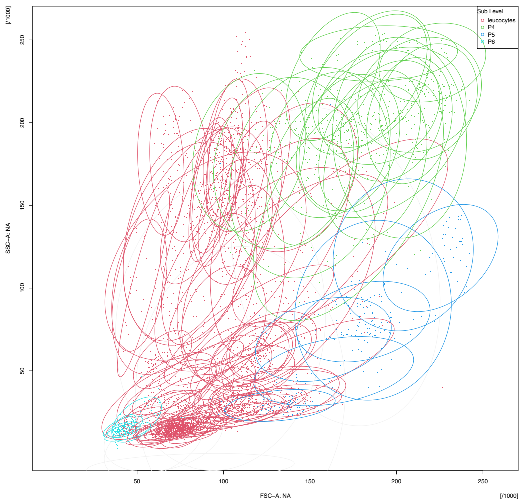

1.all_leukocytes

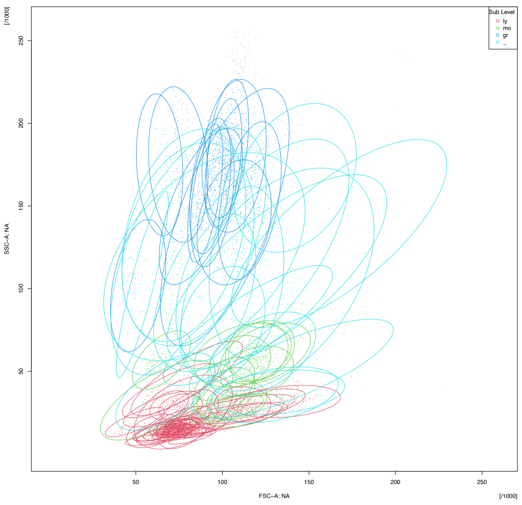

1.1.all_leukocytes_lymphocytes

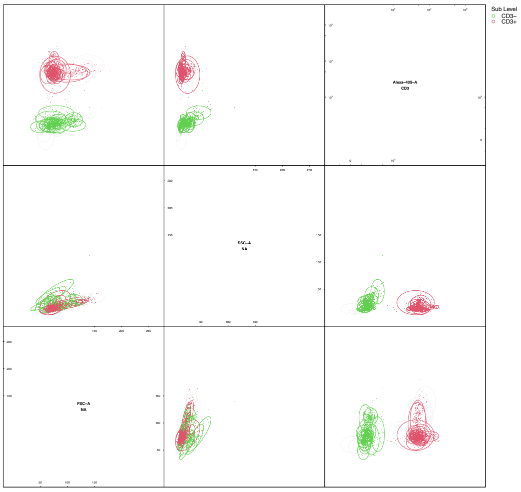

Lymphocytes CD3 negative

1.1.1.all_leukocytes_ly_CD3^{-}

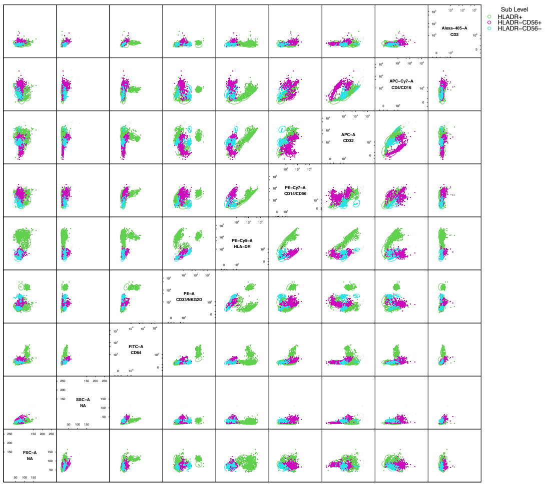

1.1.1.1.all_leukocytes_ly_CD3^{-}_HLADR^{+}

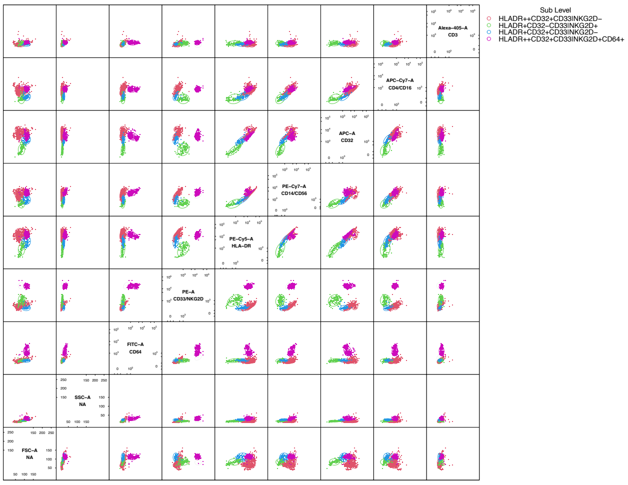

1.1.1.2.all_leukocytes_ly_CD3^{-}_HLADR^{-}CD56^{+}

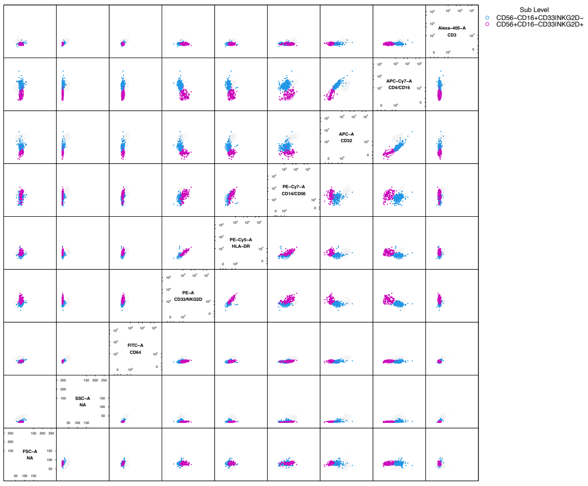

1.1.1.3.all_leukocytes_ly_CD3^{-}_HLADR^{-}CD56^{-}

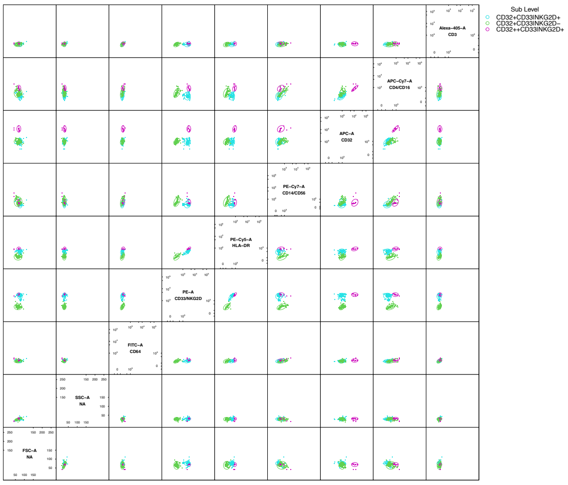

Lymphocytes CD3 positive

1.1.2.all_leukocytes_ly_CD3^{+}

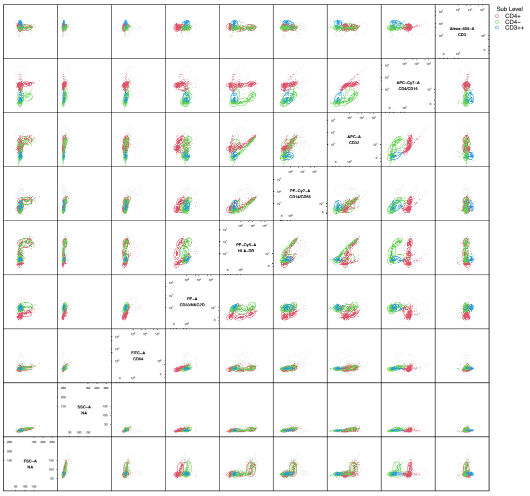

1.1.2.1.all_leukocytes_ly_CD3^{+}_CD4^{+}

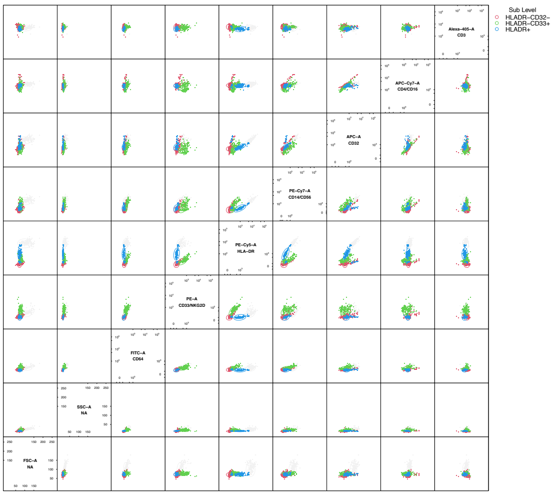

1.1.2.1.all_leukocytes_ly_CD3^{+}_CD4^{-}

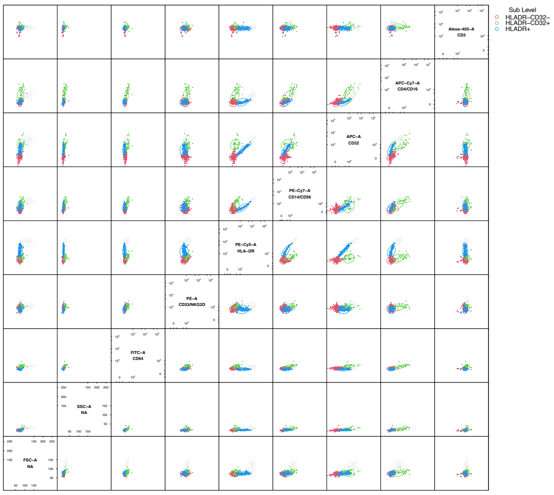

Monocytes

1.2.all_leukocytes_mo

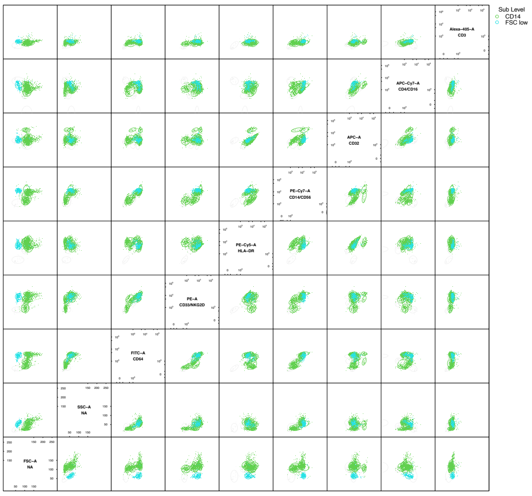

1.2.1.all_leukocytes_mo_CD14

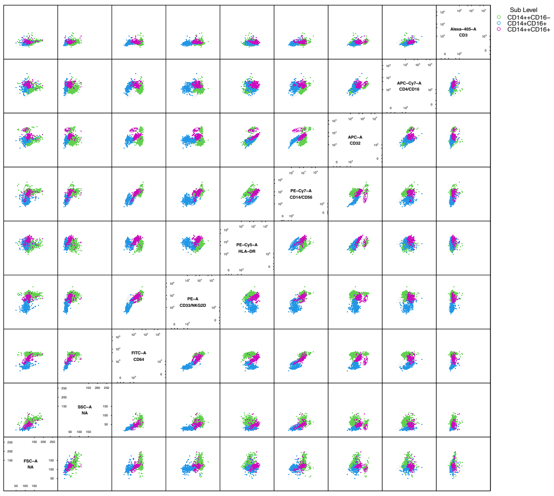

1.2.1.2.all_leukocytes_mo_CD14_CD14^{+}CD16^{+}

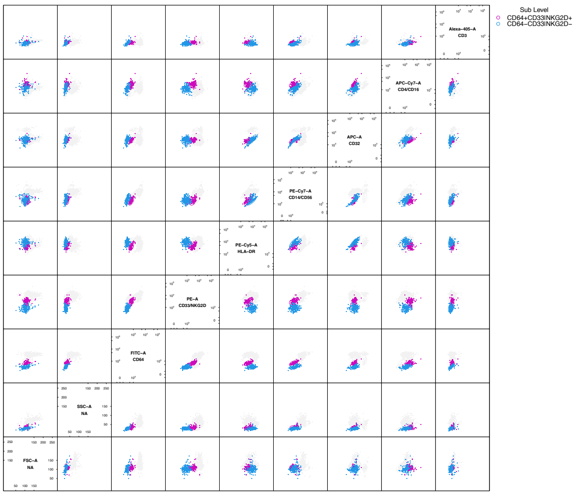

Granulocytes

1.3.all_leukocytes_granulocytes

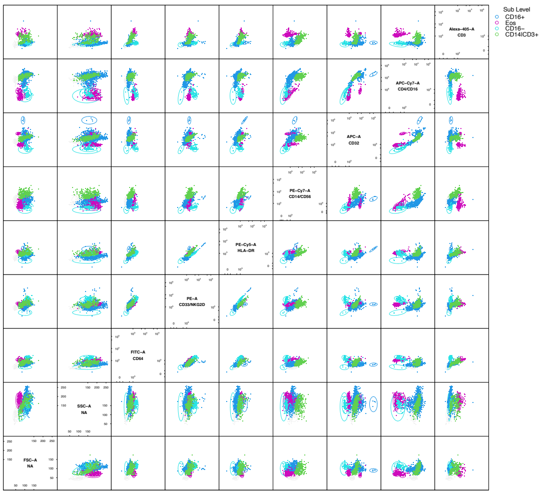

Supplement: Supplementary file 1 [file jcm-12-04359-s001.zip › Figure S1 Annotation of meta-clusters.pdf]
