# Supplementary material for: Intestinal Microbiota Reduction Followed by Fasting Discloses Microbial Triggering of Inflammation in Rheumatoid Arthritis
Source: J Clin Med. 2023 Jun 28;12(13):4359. doi: 10.3390/jcm12134359 (PMC10342944; doi:10.3390/jcm12134359)
Supplement: Supplementary file 1 [file jcm-12-04359-s001.zip › Figure S2 Improvement of disease activity.pdf]

Figure S2

Improvement of disease activity

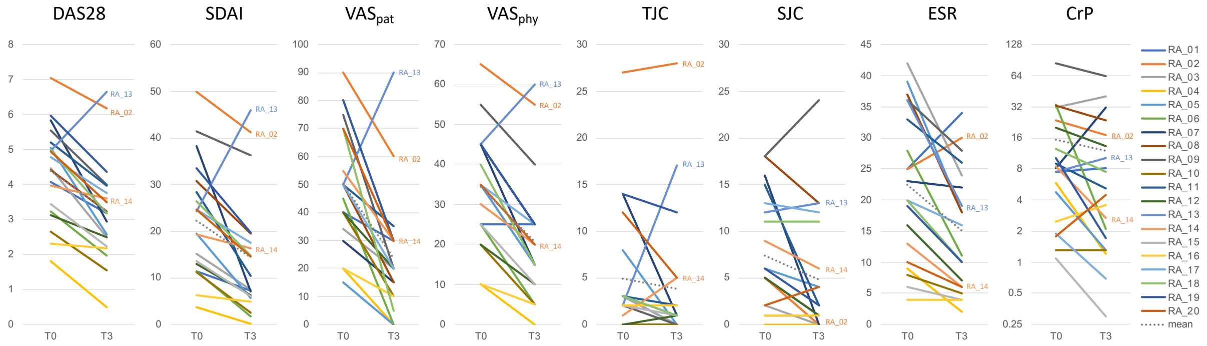

p=0.00001 § p=0.0006 § p=0.00002 § p=0.000002 § p=0.049* p=0.014* p=0.0003 § p=0.083§

§ t-test, paired
* Wilcoxon signed rank test

Legend Figure S2

Improvement of disease activity (DAS28, SDAI) and contributing parameters:

Improvement in each RA patient is displayed between T0 and T3 for the overall disease activity score DAS28, SDAI and the contributing parameters of patient ($_{pat}$) and physician ($_{phy}$) assessment of disease activity (VAS=visual analog scale from 0=no activity to 100=maximum conceivable activity), tender joint count (TJC) and swollen joint count (SJC) from 28 joints indicated in figure 1, erythrocyte sedimentation rate (ESR) and C-reactive protein (CrP). Significance of change is indicated by p-values (§=paired t-test; *=Wilcoxon signed-rank test) and non-responders according to EULAR criteria[32] were labelled separately with RA_02, RA_13 and RA_14.
