# Supplementary material for: Intestinal Microbiota Reduction Followed by Fasting Discloses Microbial Triggering of Inflammation in Rheumatoid Arthritis
Source: J Clin Med. 2023 Jun 28;12(13):4359. doi: 10.3390/jcm12134359 (PMC10342944; doi:10.3390/jcm12134359)
Supplement: Supplementary file 1 [file jcm-12-04359-s001.zip › Figure S3 non-classical monocytes.pdf]

Figure S3:

Non-classical monocytes increase in virtually all RA patients at T1 and T2:

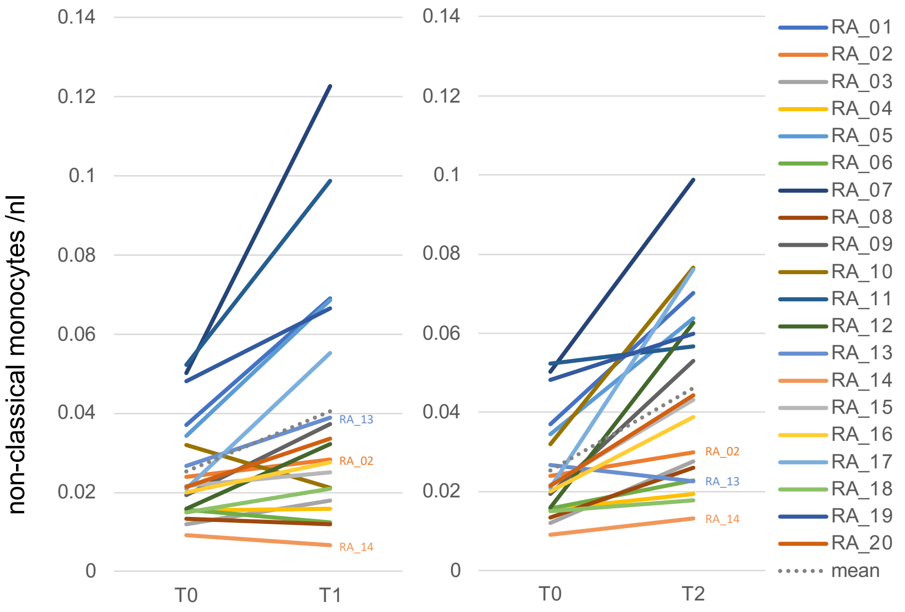

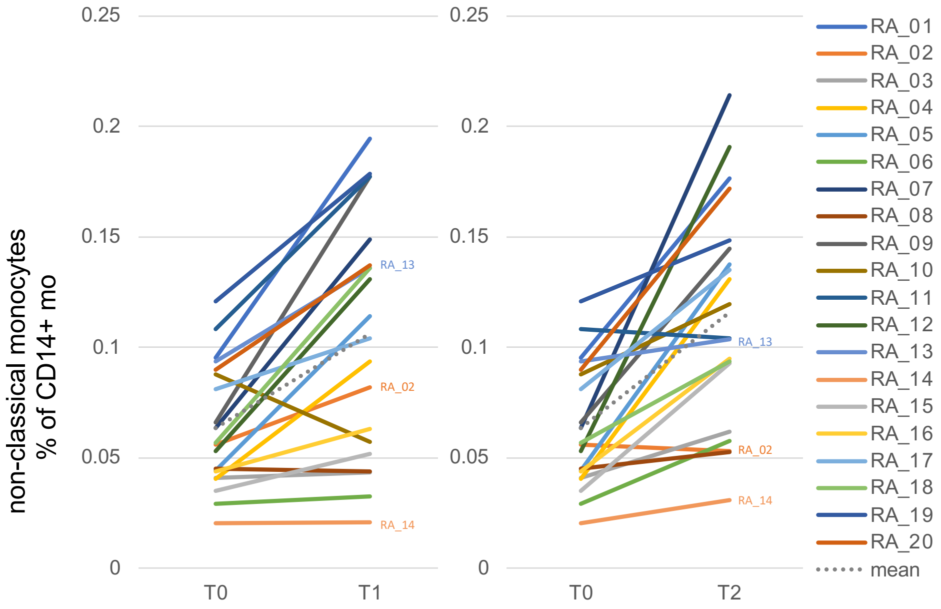

Legend Figure S3

Non-classical monocytes were determined by CD14 and CD16 staining. In RA patients, there was an increase in the absolute and relative number of non-classical monocytes, which in average became more dominant over time. Non-responders according to EULAR criteria[32] were labelled separately with RA_02, RA_13 and RA_14.
