# Supplementary material for: Intestinal Microbiota Reduction Followed by Fasting Discloses Microbial Triggering of Inflammation in Rheumatoid Arthritis
Source: J Clin Med. 2023 Jun 28;12(13):4359. doi: 10.3390/jcm12134359 (PMC10342944; doi:10.3390/jcm12134359)
Supplement: Supplementary file 1 [file jcm-12-04359-s001.zip › Legends Tables S1-S7.pdf]

Table S1: Cytometric staining matrix

Legend Table S1

Staining matrix of the antibodies with corresponding fluorochrome conjugates in the ten different staining cocktails SC-1 to SC-10.

SC-1: Staining of the leucocyte major groups

SC-2: Differential staining for immune activation processes involving FCGR1A (CD64), SIGLEC-3 (CD33), KLRK1 (NKG2D), HLA-DR and FCGR2A (CD32) predominantly on monocytes and NK-cells

SC-3: Differential staining for immune activation processes involving NKR2B4 (CD244), the scavenger receptor for the hemoglobin-haptoglobin complex (CD163), PTGDR2 (CRTH2), CXCR2 (CDw128b) and CCR2 predominantly on monocytes, neutrophils, NK-cells and CD8+ T-cells

SC-4: Differential staining for immune activation processes involving CR1 (CD35), IFNGR1 (CD119), LAMP1 (CD107a), TNFR2 (CD120b) predominantly on monocytes, neutrophils, NK-cells, CD8+ T-cells

SC-5: Differential staining for immune activation processes involving complement regulatory protein CD46, C5aR1 (CD88), CR2 (CD21) and CD55 predominantly on monocytes and neutrophils

SC-6: Differential staining for immune activation processes involving the PTPRC isoform CD45RA, CXCR4, SELL (CD62L), CCR7 and CXCR3 predominantly on T- and B-cells

SC-7: Differential staining for immune activation processes involving CLEC4C (BDCA-2), CD1c, HLA-DR and ITGAX (CD11c) predominantly on dendritic cells

SC-8: Differential staining for immune activation processes involving SDC1 (CD138), cyclic ADP ribose hydrolase (CD38), HLA-DR, CLEC2C (CD69) and MS4A1 (CD20) predominantly on T- and B-cells

SC-9: Differential staining for immune activation processes involving TNFRSF4 (CD134), ICOS, CD40LG (CD154), CLEC2C (CD69) and IL2RA (CD25) predominantly on T- and B-cells

SC-10: Differential staining for immune activation processes involving CD28, PECAM1 (CD31), PTPRC isoform CD45RA, CLEC2C (CD69) and CTLA4 (CD152) predominantly on T-cells

Table S2: Comparison of 16S sequencing results from RA and MetS stool samples

Legend Table S2

After BlastN sequence analysis and typing, the 16S sequences were quantitatively converted into frequencies for each species per sample. For each species and parent frequency in genus, family, order,

class and phylum group, comparisons were made between RA and MetS at T0 (A), T3 (B), between RA T0 and T3 (C), MetS T0 and T3 (D) and between all RA and all MetS samples (E). Each table indicates, from phylum to species, the frequency of patients in % (green), the mean frequency of microbes in % (blue), the significance level in % from 0 to 5 (red) and the magnitude of the difference in $\log_2$((mean frequency of microbes in patient group1)/(mean frequency of microbes in patient group2)) (shading in yellow for values >0, in purple for <0; species that were detected in only one group were assigned the values 10 or −10). Correlation with BMI is indicated with * (R>0.4) and § (R<−0.4) .

Table S3: Species reported in septic arthritis or associated with RA:

Legend Table S3

This table summarises species exclusive to RA that have been reported in septic arthritis or associated with RA. Literature references are given with PMID numbers and titles. Each species is grouped by genus, family, order, class and phylum, together with the results of the RA T0 vs. T3 t-test, the maximum frequencies at T0 and T3, the number of positive samples and the immediate frequencies per sample examined before and after fasting.

Table S4: Comparison of bacterial pathway scores from RA and MetS stool samples.

Legend Table S4

Similar to supplementary table 1, the scores for each pathway and sample were compared between RA and MetS at T0 (A), T3 (B), between RA T0 and T3 (C), MetS T0 and T3 (D) and between all RA and all MetS samples (E). Each table indicates, from hierarchy 1 to 6 and individual pathways the frequency of patients in % (green), the mean frequency of hierarchy/pathway in % (blue), the significance level in % from 0 to 5 (red) and the magnitude of the difference in $\log_2$((mean frequency of hierarchy or pathway in patient group1)/(mean frequency of hierarchy or pathway in patient group2)) (shading in yellow for values >0, in purple for <0; hierarchies/pathways that were detected in only one group were assigned the value 10 or −10).

Table S5: Species-specific contribution to quantitative changes in pathway hierarchy 1 between T0 and T3:

Legend Table S5

The pathway activities of the individual species were compiled per patient and the pathway scores of the individual H1 hierarchical categories (Activation-Inactivation-Interconversion, Biosynthesis, Degradation, Detoxification, Energy-Metabolism, Macromolecule-Modification, Transport pathways) were summed up per species and patient. Changes due to fasting were calculated in each H1 category as the difference between T3 and T0 for each species in each patient and for each species as the mean of all RA patients. The sum of the means of all H1 categories per species determined the order for sorting the species, so that the microbes that on average contributed most to the increase in pathway activity were at the top and those that contributed most to the decrease were at the bottom. Comparison between RA patients indicated that the majority of increases (red) were in the upper ranks and decreases (blue) in the lower ranks, but there were also patient specific differences in the increase and decrease of individual species. **(A)** changes in RA; **(B)** changes in MetS.

Table S6: Comparison of ITS and 18S sequencing results from RA and MetS stool samples.

Legend Table S6

After BlastN sequence analysis and typing, the ITS **(A)** and 18S sequences **(B)** were quantitatively converted into frequencies for each species per sample. For each species and each parent in the hierarchical groups H1 to H18, comparisons were made between RA and MetS at T0, at T3, between RA T0 and T3, MetS T0 and T3, and between all RA and all MetS samples. Each table indicates from H1 to species the frequency of patients in % (green), the mean frequency of microbes in % (blue), the significance level in % from 0 to 10 (red) and the magnitude of the difference in $\log_2$((mean frequency of eukaryotic species in patient group1)/(mean frequency of eukaryotic species in patient group2)) (shading in yellow for values >0, in purple for <0; species that were detected in only one group were assigned the values 10 or −10). **(C)** The frequencies of eukaryotic species in 18S and ITS sequencing at T0 and T3 were summarised for each patient and the two groups RA and MetS were compared. All species that differed in the Welch test ($p<0.1$) or were present only in MetS (at least in 2 patients) or only in RA (at least 3 patients) were selected. This selection resulted in a higher frequency and number of eukaryotic species in RA compared to MetS, which were significant in the sum of all 18S species ($p<0.03$) and all ITS species ($p<0.004$) as well as in their combination ($p<0.0007$).

Table S7: Estimation of fasting-induced endogenous cortisol as a dose equivalent of prednisolone:

Legend Table S7:

This table summarizes and extends the results published by Frazer et al. on endogenous cortisol production in 4 patients with RA before and after fasting (AUC 20-08 = area under the curve between 8 p.m. and 8 a.m.)[25]. As reported, they observed an increase in endogenous cortisol only during the night. For calculation of the dosis produced in mg, the half-life (t1/2) and volume of distribution (Vd) of cortisol in vivo are used for low and high dose treatment according to recently published values[53]: 1) low dose: t1/2=1.82 hours; Vd=32.98 litres; 2) high dose: t1/2=2.0 hours; Vd=50.33 litres. The increase in AUC in each patient after fasting is converted to an amount of additional cortisol production in mg/day for both dosing assumptions and then converted to the corresponding amount of prednisolone in mg/day. This results in an average additional amount of prednisolone of about 2 mg (1.67 mg to 2.31 mg) per day during fasting, which, based on practical experience of RA treatment with glucocorticoids, suggests, if at all, a much smaller clinical improvement than that observed.
